# Pharmacokinetic Analysis of ^111^In-Labeled Liposomal Doxorubicin in Murine Glioblastoma after Blood-Brain Barrier Disruption by Focused Ultrasound

**DOI:** 10.1371/journal.pone.0045468

**Published:** 2012-09-18

**Authors:** Feng-Yi Yang, Hsin-Ell Wang, Ren-Shyan Liu, Ming-Che Teng, Jia-Je Li, Maggie Lu, Ming-Cheng Wei, Tai-Tong Wong

**Affiliations:** 1 Department of Biomedical Imaging and Radiological Sciences, National Yang-Ming University, Taipei, Taiwan; 2 Department of Nuclear Medicine, National Yang-Ming University Medical School, Taipei Veterans General Hospital, Taipei, Taiwan; 3 Drug Delivery Lab, Biomedical Technology and Device Research Laboratories, Industrial Technology Research Institute, HsinChu, Taiwan; 4 Department of Neurosurgery, Neurological Institute, Taipei Veterans General Hospital, Taipei, Taiwan; Faculté de médecine de Nantes, France

## Abstract

The goal of this study was to evaluate the pharmacokinetics of targeted and untargeted ^111^In-doxorubicin liposomes after these have been intravenously administrated to tumor-bearing mice in the presence of blood-brain barrier disruption (BBB-D) induced by focused ultrasound (FUS). An intracranial brain tumor model in NOD-*scid* mice using human brain glioblastoma multiforme (GBM) 8401 cells was developed in this study. ^111^In-labeled human atherosclerotic plaque-specific peptide-1 (AP-1)-conjugated liposomes containing doxorubicin (Lipo-Dox; AP-1 Lipo-Dox) were used as a microSPECT probe for radioactivity measurements in the GBM-bearing mice. Compared to the control tumors treated with an injection of ^111^In-AP-1 Lipo-Dox or ^111^In-Lipo-Dox, the animals receiving the drugs followed by FUS exhibited enhanced accumulation of the drug in the brain tumors (*p*<0.05). Combining sonication with drugs significantly increased the tumor-to-normal brain doxorubicin ratio of the target tumors compared to the control tumors. The tumor-to-normal brain ratio was highest after the injection of ^111^In-AP-1 Lipo-Dox with sonication. The ^111^In-liposomes micro-SPECT/CT should be able to provide important information about the optimum therapeutic window for the chemotherapy of brain tumors using sonication.

## Introduction

Malignant brain tumors remain difficult to treat using chemotherapy because the blood-tumor barrier (BTB) limits the amount of potent agents that can be delivered to the tumor; the result is that the drug is usually unable to reach a therapeutic level. Despite the fact that the BTB is in itself more permeable than the blood–brain barrier (BBB), therapeutics are rarely effective in patients with brain tumors because the selective permeability of the BTB still blocks many antitumor agents and stops them approaching their target [1]. Glioblastoma multiforme (GBM) is one of the most common forms of glioma and is hard to treat completely by surgical resection due to the diffuse nature of the glioma. As a result, residual microscopic tumor cells usually need to be eliminated by additional chemotherapy or radiotherapy [2].

Liposomes are polymeric nanoparticles that consist of phospholipid bilayer structure and can be used to effectively encapsulate chemotherapeutic agents. These encapsulated agents exhibit improved pharmacokinetics, better biodistribution, and lower tissue toxicity. Long-circulating liposomes were found to accumulate in tumor by enhanced permeability and retention (EPR) effect. They are delivered mainly to the regions of interest and the above properties enhance the therapeutic effectiveness of a given drug [3], [4], [5]. One negative aspect of traditional liposomes is that they are easily taken up by reticuloendothelial system (RES). Polyethylene glycol (PEG) conjugated liposomes were developed to evade this rapid clearance by the RES and allow long-term circulation of the liposomal drugs in order to prolong the period of treatment [6], [7]. Compared to conventional liposome formulations, the PEGylated liposomal formulation of doxorubicin produces a marked improvement in antitumor effects, enhances cancer cell targeting and improves treatment efficacy [8], [9].

It has been reported that human brain tumor cell lines overexpress high plasma membrane interleukin-4 receptors (IL-4R) compared with normal brain tissue [10], [11]. IL-4R-targeted cytotoxin has been shown to mediate a remarkable antitumor effect in immunodeficient xenograft models of human GBM tumors [12]. These findings show that therapeutic agents that bind to IL-4R allow selective drug delivery that aids tumor treatment [13]. We designed as a ligand for the atherosclerotic plaque-specific peptide-1 (AP-1) which 9 amino acids sequence was selected from phage display libraries. The AP-1 peptides can locate atherosclerotic plaque tissue and bind to the IL-4 receptor, since it has the same binding motif to the IL-4 protein [13]. Our previous studies have demonstrated that focused ultrasound (FUS) not only significantly increases the permeability of the BBB at the sonicated site, but also significantly elevates the lesion-to-normal brain ratio in the focal region [14], [15], [16]. Another study has shown that FUS is able to promote accumulation of liposomal doxorubicin (Lipo-Dox) at the therapeutic levels [17].

However, up to the present, quantitative data exploring the pharmacokinetics of the targeted drug after sonication are not yet available. In this study, we applied micro-SPECT imaging in a GBM-bearing animal model system to evaluate the pharmacokinetics of targeted and untargeted ^111^In-doxorubicin liposomes with or without FUS sonication.

## Results

**Figure 1:**
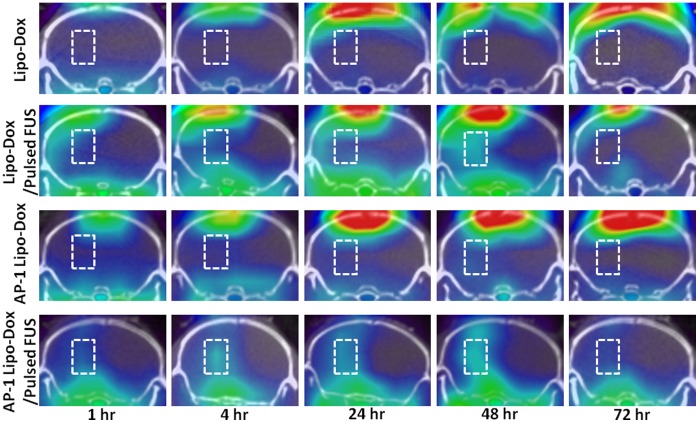
Micro-SPECT/CT axial images of GBM-bearing mice at 1, 4, 24, 48 and 72 hr after intravenous injection of 500 µCi/0.1 mL of targeted or untargeted ^111^In-doxorubicin liposomes. Brain tumors and contralateral normal brain are in the left and right hemispheres, respectively. The regions-of-interest (ROI) for tumors are indicated by rectangles in the left hemispheres. There was a clear radioactivity at 1, 4, 24, and 48 hr in the ROI of mice treated with AP-1 Lipo-Dox followed by pulsed FUS (bottom row).

No radioactivity was detected in the tumors after administration of ^111^In-Lipo-Dox or ^111^In-AP-1 Lipo-Dox alone (Fig. 1). A significant extravasation of radioactivity can be seen in the BBB opening regions of the left hemisphere. In the microSPECT/CT scan of the GBM-bearing mice, high contrast between the sonicated tumor and unsonicated tumor can be seen at 4, 24 and 48 hr after the injection of ^111^In-Lipo-Dox or ^111^In-AP-1 Lipo-Dox, especially for ^111^In-AP-1 Lipo-Dox. Moreover, BBB disruption in the left sonicated tumor can be clearly seen in terms of uptake of ^111^In-Lipo-Dox or ^111^In-AP-1 Lipo-Dox at 48 hr after administration.

**Figure 2:**
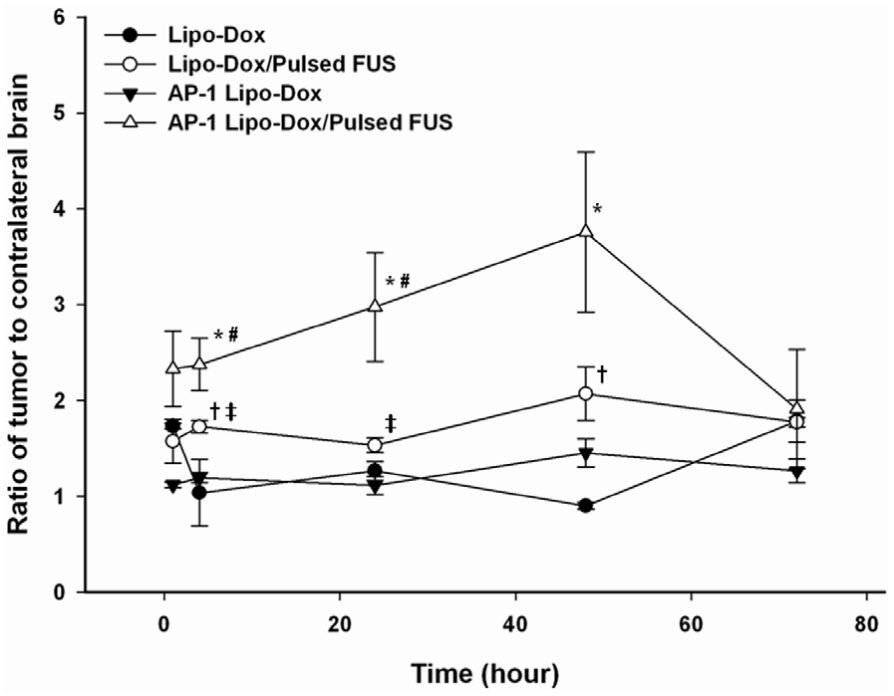
Pharmacokinetics of the ^111^In-doxorubicin liposomes in GBM-bearing mice determined by micro-SPECT/CT. Tumor-to-contralateral brain ratios for ^111^In-doxorubicin liposomes in the GBM-bearing mice were derived from the dynamic micro-SPECT/CT images after intravenous injection followed by sonication or nonsonication. * and # denote a significant difference compared with AP-1 Lipo-Dox and Lipo-Dox plus FUS, respectively (* and #, *p*<0.05). + and ‡ denote a significant difference compared with Lipo-Dox and AP-1 Lipo-Dox, respectively (+ and ‡, *p*<0.05).

**Figure 3:**
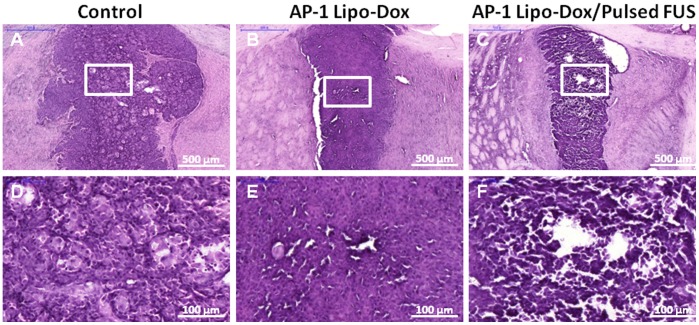
Twelve days after tumor implantation, three mice each form the control group, the targeted Lipo-Dox without pulsed FUS group, and the targeted Lipo-Dox with pulsed FUS group were sacrificed. Sections of the tumors were collected for hematoxylin and eosin histological examination. (scale bar = 500 µm [a-c]; scale bar  = 100 µm [d-f]).

Figure 2 reveals that the tumor-to-contralateral brain ratios derived from the dynamic SPECT images of the tumors are significantly greater after FUS sonication. However, no significant differences were found for the ratios of unsonicated tumors treated with ^111^In-Lipo-Dox or ^111^In-AP-1 Lipo-Dox between various time points. The tumor-to-contralateral brain ratios after ^111^In-Lipo-Dox or ^111^In-AP-1 Lipo-Dox administration show a peak value of about 2.1 or 3.8, respectively, at 48 hr after intravenous injection followed by sonication. Furthermore, the ratio showed a rapid decrease beyond 48 hr after administration of ^111^In-AP-1 Lipo-Dox plus sonication treatment. Regardless of targeted or untargeted ^111^In-Lipo-Dox, the ratios showed no difference between the sonicated tumor and unsonicated tumor at the 72 hr time point. The histology images (Fig. 3) showed that at 12 days after implantation there was local displacement and widening of intercellular gaps in the tumor tissues treated with AP-1 Lipo-Dox followed by sonication relative to the control tumors or tumors treated with AP-1 Lipo-Dox alone.

**Figure 4:**
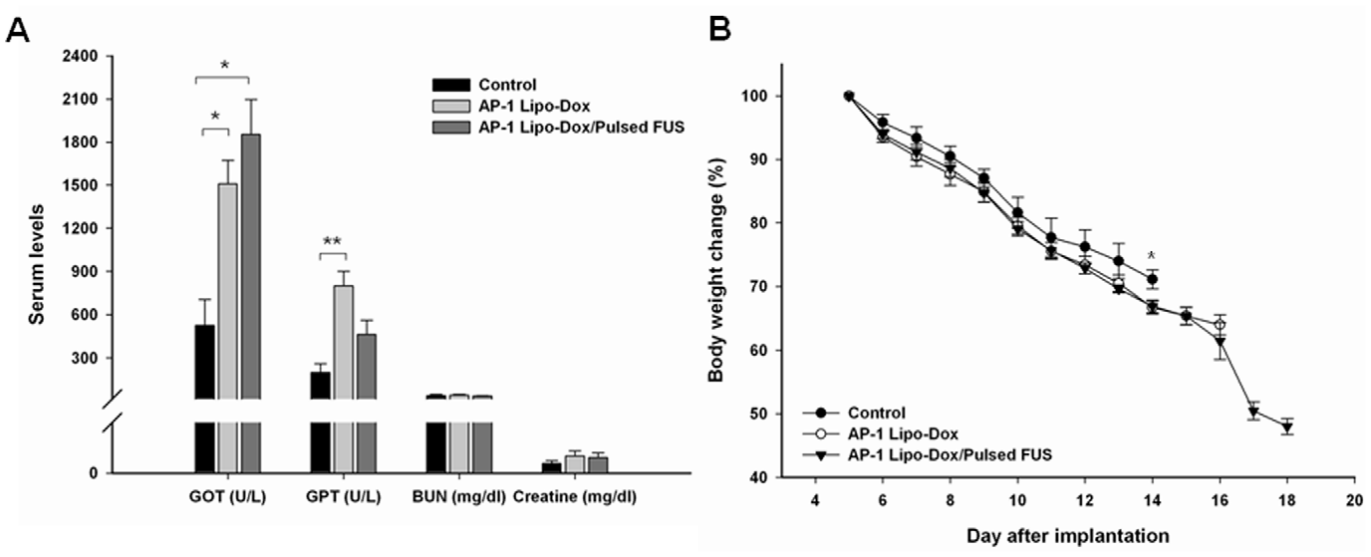
Systemic toxicity of AP-1 Lipo-Dox in tumor-bearing mice. (a) Serum chemistry of mice treated with AP-1 Lipo-Dox without or with sonication. The control tumor group received phosphate-buffered saline. Mice received treatments on days 5 and 9 after implantation. All animals were sacrificed for toxicity evaluation on day 12 after implantation. GOT = glutamic oxaloacetic transaminase, GPT = glutamic pyruvic transaminase, BUN = blood-urea nitrogen; ^*^ *p*<0.05, ^**^ *p*<0.01. (b) Mean body weights (relative to day 5) of tumor-bearing mice treated with AP-1 Lipo-Dox with or without sonication.

Dox treatment results in severe irreversible cardiomyopathy and hepatotoxicity in humans [18]. Figure 4a shows the effect of Dox on biochemical changes in the serum of mice. Treatment of mice with AP-1 Lipo-Dox alone significantly elevated the activities of glutamic-oxaloacetic transaminase (GOT) and glutamic-pyruvic transaminase (GPT), indicating drug-induced hepatic toxicities in these animals. The activity of GOT was also significantly higher in mice treated with AP-1 Lipo-Dox plus sonication. However, there was no significant difference in GPT activity between the control group and the mice treated with AP-1 Lipo-Dox with sonication. None of the mice treated with AP-1 Lipo-Dox, without or with sonication, had abnormal serum chemistry for blood-urea nitrogen or creatine levels, indicating the absence of renal toxicity. Both treatment protocols effected a slight decrease in body weight compared to the untreated controls (Fig. 4b). There was a significant decrease in body weight on day 14 in the AP-1 Lipo-Dox-alone group after tumor implantation (*p*<0.05). However, treatment with AP-1 Lipo-Dox plus sonication was not associated with a statistically significant drop in body weight.

**Figure 5:**
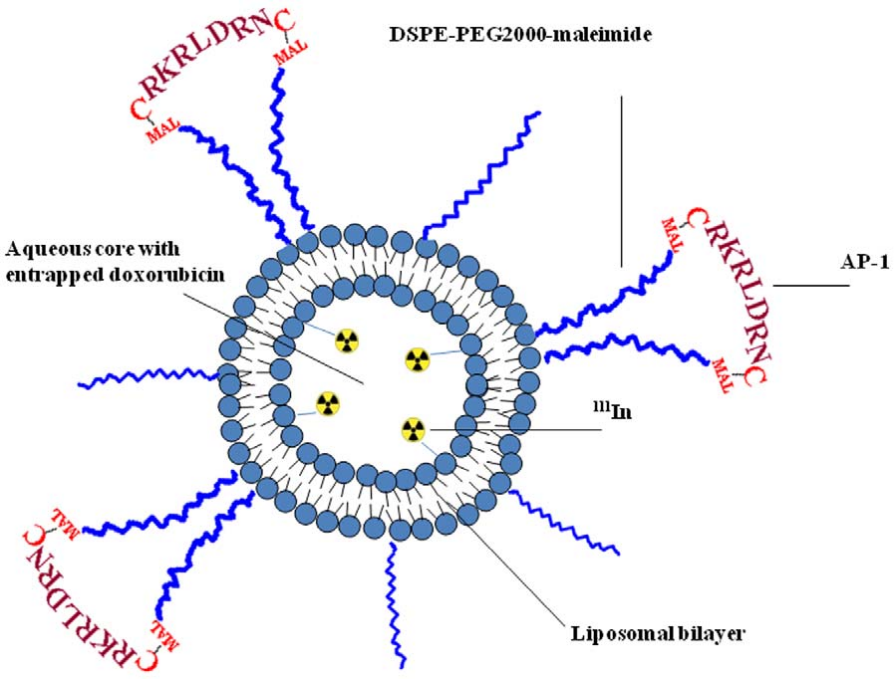
Schematic diagram for the ^111^In-AP-1 Lipo-Dox. Liposomes were prepared containing maleimide-functional polyethylene glycol chains. The maleimide was used to attach the AP-1 peptide through the thiol group on a cystine. ^111^In label was introduced into the liposomes. Therapeutic liposomes were loaded with Dox. DSPE-PEG2000 = 1,2-distearoyl-*sn*-glycero-3-phosphoethanolamine-N-[methoxy(polyethylene glycol)-2000], MAL = maleimide.

**Table 1:** The composition and properties of prepared Lipo-Dox and AP-1 Lipo-Dox.

Formulation	Composition(molar ratio)	Particle size (nm)	Polydispersity index	Zeta potential (mV)	Dox concentration(mg/ml)
Lipo-Dox	6∶4∶0.5	101.0±24.5	0.089	−23.9±4.8	1.87
AP-1 Lipo-Dox	6∶4∶0.5∶0.15	116.1±30.3	0.124	−26.6±3.7	1.66

## Discussion

FUS is a technique that enables to temporarily disrupt the BBB, and has been investigated in a number of papers for its potential to promote penetration of larger molecules into brain tumors [14], [16]. In addition, macromolecular agents or nanoparticles attached to targeting ligands can be taken up along, enabling intrinsically BBB-impermeable compounds to enter the BBB [19], [20]. However, to our knowledge, the pharmacokinetics after injection of active targeting nanoliposome contained Dox with BBB disruption induced by FUS has not been reported previously. Therefore, we investigated the pharmacokinetics after injection of ^111^In-Lipo-Dox and ^111^In-AP-1 Lipo-Dox with or without BBB disruption using noninvasive FUS technology.

**Figure 6:**
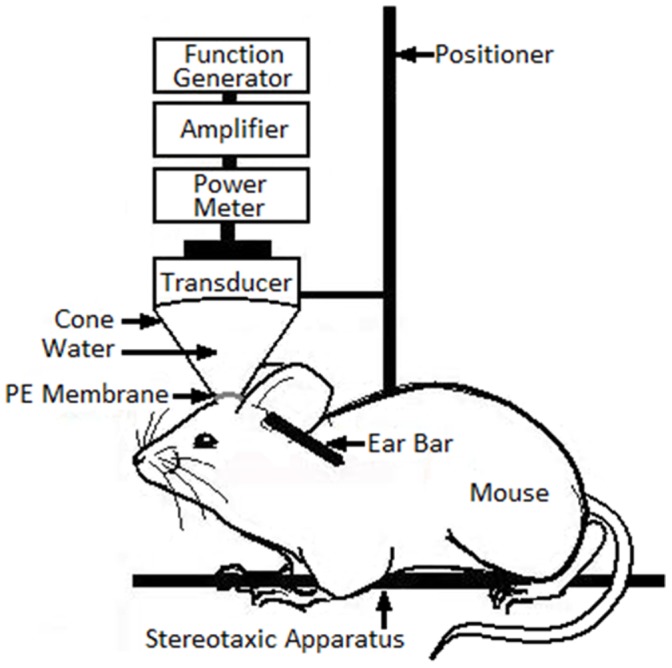
Schematic diagram of the experimental setup for blood-brain barrier opening. The positioner was also mounted on a stereotaxic apparatus. PE, polyurethane.

Dynamic SPECT imaging demonstrated significant elevations in the tumor-to-normal brain ratio of targeted Lipo-Dox followed by sonication as compared to targeted Lipo-Dox alone under the same experimental conditions (Fig. 2). The tumor-to-contralateral brain ratios after injection of ^111^In-Lipo-Dox or ^111^In-AP-1 Lipo-Dox reached a maximum value at 48 hr, with the FUS peak values being more than twice as great as that without the FUS treatment, but these values declined to the levels of the unsonicated tumor at 72 hr. The cytotoxin (IL-4(38-37)-PE38KDEL), is a fusion protein containing translocation and enzymatic domains of Pseudomonas exotoxin and a circularly permuted human IL-4. Although the receptor for interleukin-4 (IL-4R) is highly expressed on solid human cancer cells, its internalization function is still unclear. On the basis of our results, the ability to actively target nanoparticles into the brain tumor would seem to be significantly elevated by sonication. It seems likely that FUS is able to enhance the concentration of targeting nanoparticles in the brain tumor tissue due to BBB disruption. Subsequently, the ligand has a high binding capacity to the tumor cells. However, the effect of FUS on tumor cell biomarkers is still unknown and further investigation is needed. By combining biology-directed and physically-assisted methods, hopefully this synergistic technology can safely produce a high tumor-to-normal brain ratio specifically in the brain tumor without causing extra toxicity to normal brain tissue and other systemic side effects.

It has been proposed that pulsed FUS generates acoustic radiation forces that occur due to absorption of energy in the focal zone, thereby producing local displacements in the tissues in and around the focal region (Fig. 3c, f) [21]. It has been shown previously that sonication induces widening of the intercellular gaps [22]. This technology also reduces a high interstitial fluid pressure in tumors. The opening up of the intercellular spaces in the tumors by FUS seems to enhance nanoparticle transport in the interstitium and, simultaneously, lower interstitial fluid pressure for improved extravasation.

Figure 4a reveals that AP-1 Lipo-Dox with sonication is able to decrease the AP-1-Lipo-Dox-induced elevation of GPT activity. Body weight was monitored in order to compare the systemic toxicities of AP-1 Lipo-Dox without and with sonication (Fig. 4b). Both treatments had little effect on body weight, although there was a significant reduction in body weight at day 14 following implantation with AP-1 Lipo-Dox treatment alone. Thus, AP-1 Lipo-Dox with sonication does not confer additional toxicity compared to AP-1 Lipo-Dox alone. Our results cannot prove unequivocally that FUS is able to reduce any systemic toxicity and there is a need for further investigation of the biodistribution of active targeted nanoliposomes loaded with Dox when there is FUS exposure.

This study demonstrates that ligand-conjugated liposomal technology assisted by FUS is able to increase the uptake of therapeutic agents in brain tumors and elevate the tumor-to-contralateral brain ratio. Combining targeted Lipo-Dox with sonication gives rise to a unique pharmacokinetic profile and knowledge of this profile can serve as a starting point for the development of synergistic technology that can be used for preclinical applications.

## Materials and Methods

### Glioma Xenograft Model and Animal Preparation

Male 6-week to 8-week-old NOD-*scid* mice were anesthetized by an intraperitoneal administration of pentobarbital at a dose of 40 mg/kg of body weight. All procedures were performed according to guidelines and approved by the Animal Care and Use Committee of the National Yang-Ming University. Mice were shaved on the head above the nape of the neck, scrubbed with betadine/alcohol, and immobilized in a Cunning-ham Mouse/Neonatal Rat Adaptor stereotactic apparatus (Stoelting, Wood Dale, IL, USA). A 5-mm skin incision was made along the sagital suture and a burr hole drilled into the skull. Human brain malignant glioma cells (GBM8401) were obtained from the Bioresource Collection and Research Center of Taiwan [23]. GBM8401 cells were transformed with Luciferase gene (GBM8401-luc) and then 2×10^5^ GBM8401-luc cells in 2 µL culture medium were injected into the brains of these mice. The glioma cells were stereotactically injected into a single location in the left hemisphere (0.14 mm anterior and 2.0 mm lateral to the bregma) of each mouse at a depth of 3.5 mm from the brain surface. Next, the burr holes in the skull were sealed with bone wax and the wound was flushed with iodinated alcohol. Bioluminescence imaging was used to determine that a tumor was established.

### Radiolabeling of ^111^In-Oxine

Oxine was labeled with ^111^In as previously described by Chow et al. [24], [25]. In total, 15 mL of 68 mM 8-hydroxyquinoline (oxine; Sigma-Aldrich Co.) in ethanol were added to 10 µL of ^111^In (indium chloride in 0.05 M HCl; 0.01–1 mCi, Perkin Elmer) in 400 µL of 0.1 M sodium acetate buffer (pH 5.5) and then incubated at 50°C for 20 min. The lipophilic components were extracted with chloroform and then dried using a rotary evaporator. The labeling efficiency with ^111^In-oxine was determined by the instant thin-layer chromatography (ITLC) method. ITLC was performed on a silica-gel-impregnated glass fiber sheet (ITLCTM SG, Pall Corporation) using ethanol as the developing agent. The radiochemical yield was generally greater than 90% ^111^In-oxine.

### Preparation of ^111^In-Doxorubicin Liposomes

Lipo-Dox was prepared using a solvent injection method plus remote loading procedures. Due to the presence of a thiol group on each cystine of the AP-1 peptide (CRKRLDRNC), it is possible to couple AP-1 to liposomes via the thiol-maleimide reaction. Briefly, AP-1 peptide was conjugated to 1,2-distearoyl-*sn*-glycero-3-phosphoethanolamine-N-[maleimide(polyethylene glycol)-2000] (DSPE-PEG2000-MAL, Avanti Polar Lipids) by adding AP-1 to the DSPE-PEG2000-MAL micelle solution at a 2∶1 molar ratio while mixing at 4°C overnight. The free thiol groups were measured with 5,5?-dithiobis-(2-nitrobenzoic acid) (Ellman’s reagent, Sigma-Aldrich) at 420 nm to confirm that most of the AP-1 was conjugated with DSPE-PEG2000-MAL after the reaction. AP-1-conjugated DSPE-PEG2000 was transferred into the preformed Lipo-Dox at a 1.5% molar ratio of total lipid components and incubated at 60°C for 1 h to obtain AP-1-labeled Lipo-Dox (AP-1 Lipo-Dox; Fig. 5). The resulting unconjugated Lipo-Dox and AP-1 Lipo-Dox were found to have particle diameters of 100∼120 nm, as measured by a dynamic light-scattering apparatus (Coulter N4 plus, Beckman), as well as a surface zeta potential of between –20 and –30 mV, as measured by electrophoretic light scattering (ZetaPlus, Brookhaven). The composition and properties of each preparation are given in Table 1.

The extracted ^111^In-oxine in chloroform was evaporated to dryness. This was followed by the addition of 20 µL of ethanol and 80 µL of distilled water into the vial, and then the mixture was incubated with 1.5 ml of liposomes for 30 min at 37°C. Ethylenediaminetetraacetic acid (2 mg) was then added to chelate any residual free ^111^In and to promote prompt excretion after intravenous injection. The labeling of ^111^In within the liposomes (6% PEG) was assayed by loading a 100-µL sample onto a Sephadex G-50 Fine (40×8 mm) column, followed by elution with normal saline. The labeling efficiency was determined by dividing the radioactivity from doxorubicin liposome fractions by the total amount loaded. The radioactivity of each fraction was measured using either a dose calibrator (CRC-15R, Capintec; Bioscan) or a gamma scintillation counter (Cobra II Autogamma; Packard). The entrapment of ^111^In was more than 90%. The labeling efficiency was 95%, the specific activity was 500 µCi/76 ng, and the radiochemistry >98%.

### Pulsed FUS System and Sonication

Pulsed FUS exposures were performed with a single-element focused transducer (A392S, Panametrics, Waltham, MA, USA); this has a diameter of 38 mm, a radius of curvature of 63.5 mm, and a resonant frequency of 1.0 MHz. The focal zone of the therapeutic transducer was in the shape of an elongated ellipsoid, with a radial diameter (–6dB) of 3 mm and an axial length (–6dB) of 26 mm. The whole transducer-driving system is similar to that used in our previous work [26], [27] (Fig. 6).

Ultrasound contrast agent (UCA) in the vasculature is served as nuclei for cavitation which is the interaction between microbubbles and ultrasound. UCA (SonoVue, Bracco International, Amsterdam, The Netherlands) was injected into the tail vein of the mice about 10 s before each sonication. This agent contains phospholipid-coated microbubbles at a concentration of 1–5×10^8^ bubbles/ml, with the bubbles having a mean diameter of 2.5 µm. The sonication was precisely targeted using a stereotaxic apparatus that utilized the bregma of the skull as an anatomical landmark. The ultrasound beam was delivered to one location in the left brain hemisphere, centered on the tumor injection site. The following sonication parameters were used: an acoustic power of 2.86 W (corresponding to a peak negative pressure of 0.7 MPa) with an injection of 300 µl/kg UCA, a pulse repetition frequency of 1 Hz, and a duty cycle of 5% (50 ms “on” and 950 ms “off”). Five days after tumor cell implantation, the GBM-bearing mice received one of the following: (1) ^111^In-Lipo-Dox, (2) ^111^In-Lipo-Dox followed by pulsed FUS, (3) ^111^In-AP-1 Lipo-Dox, (4) ^111^In-AP-1 Lipo-Dox followed by pulsed FUS.

### Micro-SPECT/CT Imaging

A FLEX Triumph™ pre-clinical imaging system (Gamma Medica-Ideas, Inc., Northridge, CA, USA) was used for the small-animal SPECT/CT image acquisition. This system applied circular scanning protocols for both SPECT and CT acquisition, with a translation stage in a variable axial imaging range. The axial field of view (FOV) for CT without stage translation was 61.44 mm. The CT system had a power-adjustable X-ray emitter ranging from 50 to 80 kVp and a microfocus (<50 µm) tube. Each mouse was injected intravenously with 500 µCi/0.1 mL of ^111^In-doxorubicin liposomes. The SPECT projection dataset was acquired using three low-energy, high-resolution pinhole collimators with a radius of rotation of 50 mm. The mice were anesthetized by inhalation of isoflurane with oxygen and then scanned by CT using 512 slides for anatomic coregistration; they then underwent a dynamic SPECT sequence involving eight frames. A total of 32 projections of 28 s were acquired over 180° and these formed a 60×60 matrix, which needed a total imaging time of 30 min per frame. The image dataset was then reconstructed using the ordered subset expectation maximization (OSEM) algorithm with standard mode parameter as provided by manufacturer. No scatter or attenuation correction was applied to the reconstructed images. A pinhole SPECT acquisition of a standard amount of radioactivity was performed as a reference for quantification and decay was corrected using radioactivity counts measured with a γ–counter (VDC-405, Veenstra Instruments, The Netherlands).

The images were viewed and quantified using AMIDE software [28] (free software provided by SourceForge). Cylindrical regions of interest (ROIs, π×(0.75)^2^×3 mm^3^) under the skull defect were manually pinpointed at the sonicated site and in the same region of the contralateral brain. The image counts within the ROIs were converted to absolute radioactivity using an efficiency factor determined from the reference standard radioactivity. The mean radioactivity within each ROI was obtained by subtracting the background radioactivity for the same size of ROI in muscle from the radioactivity of each ROI. The mean radioactivity within the blood-brain barrier disruption (BBB-D) region at each different time was determined and compared to the results obtained from the equivalent contralateral brain region.

### Histological Observations

Two tumor-bearing mice from the AP-1 Lipo-Dox or the pulsed FUS plus AP-1 Lipo-Dox treatment groups and two control mice were sacrificed 12 days after tumor implantation for histological analysis. The mice were perfused with saline and 10% neutral buffered formalin. Their brains were removed and embedded in paraffin, and then serially sectioned into 30-µm-thick slices. The slices were stained with hematoxylin and eosin in order to confirm tumor progression. The histological evaluation was carried out by light microscopy (BX61, Olympus).

### Toxicity Analysis

Five days after tumor cell implantation, the GBM-bearing mice received AP-1 Lipo-Dox or AP-1 Lipo-Dox plus FUS. The mice were anesthetized and 300 µl of blood was collected by cardiac puncture on day 12 after implantation. The serum was measured for the expression of glutamic oxaloacetic transaminase, glutamic pyruvic transaminase, blood-urea nitrogen, and creatine. All measurements were performed at the experimental animal center at National Taiwan University College of Medicine by technicians who were blinded to the experimental procedures. Four mice were assessed from each group.

### Statistical Analysis

All values are shown as means ± SEM. Statistical analysis was performed using an unpaired Student *t* test. Statistical significance was defined as a *p* value ≤ 0.05.
